# Large Scale Food Fortification (LSFF) in East, Central, and Southern Africa: Gaps, opportunities and the way forward

**DOI:** 10.1371/journal.pone.0345215

**Published:** 2026-04-10

**Authors:** Justine A. Kavle, Joseph Gaithuma, Ruth Waithira, Lidan Du-Skabrin

**Affiliations:** 1 Kavle Consulting, Westlands, Nairobi, Kenya; 2 Kavle Consulting, Washington, District of Columbia, United States of America; Indian Institute of Information Technology, INDIA

## Abstract

**Background:**

Micronutrient deficiencies affect 2 billion people globally, especially in sub-Saharan Africa, where iron, vitamin A, iodine, zinc, calcium, selenium, folate, and vitamin B12 deficiencies are common. Large Scale Food Fortification (LSFF) – addition of micronutrients to commonly consumed, industrially processed foods and condiments – can improve micronutrient intake at population level. Progress across East Central Africa Health Community (ECSA-HC) and the Southern Africa Development Community (SADC) regarding fortification of wheat and maize flour, edible oils, and sugar has been uneven. This paper discusses the current status of LSFF, challenges and opportunities to inform future LSFF programming.

**Methods:**

Qualitative situation analysis comprised of 19 key informant interviews (KIIs) with global, regional, and country stakeholders. KIIs were analyzed by themes and subthemes using Dedoose software.

**Results:**

Findings reveal wide variation in LSFF progress across ECSA-HC and SADC countries, with better institutionalized legislation, coordination, and monitoring in ECSA-HC countries than SADC countries. Key challenges are: weak enforcement of mandatory food fortification standards, little data on market reach and coverage of fortified foods and limited country capacity for monitoring LSFF programs. Opportunities to strengthen LSFF program implementation, measured by compliance and coverage of fortified foods, center on raising awareness of public health benefits of LSFF via targeting governments and consumers, engaging private sector to improve compliance with fortification standards, and incorporating small-scale producers into LSFF.

**Conclusion:**

Moving forward, investment on LSFF programming by country governments, and private sector should prioritize integrating reach and coverage indicators into existing national health surveys. Supporting public education campaigns to raise awareness of fortified foods’ health benefits, improving collaboration between government regulators and industry quality assurance personnel (i.e., small millers) through targeted fiscal policies and/or incentives as well capacity strengthening for compliance to fortification standards to attain intended nutrition outcomes for LSFF programming is sorely needed.

## Background

Globally, micronutrient deficiencies affect two billion people and if left unaddressed, can contribute to an increased risk of birth defects, poor cognitive development, and reduced adult productivity, perpetuating the intergenerational cycle of malnutrition [1]. Micronutrient deficiencies, especially iron, vitamin A, and iodine, are prevalent in sub-Saharan Africa [2], Moreover, varying severity of zinc, calcium, selenium, folate and vitamin B_12_ deficiencies are present on the African continent- largely dependent on the composition of local diets which are often lacking dietary diversity [1,3–6]. In East and Southern Africa region, anemia, as a consequence of micronutrient deficiencies and other contributory factors, affects nearly 42% of women of reproductive age (i.e., ranging from 29% in South Africa to 55% in Mozambique) [6]. Large Scale Food Fortification (LSFF) - defined as the addition of micronutrient(s) to commonly consumed, industrially processed foods and condiments - is widely regarded as a feasible and sustainable food system intervention to improve population-level micronutrient intake [7–9]. The viability of a food fortification program relies upon key components such as participation of large-scale food industries, practical and easily interpretable standards and/or regulations, the presence of low cost, efficient industry quality control and assurance(QC/QA), and government inspections – alongside ongoing epidemiological surveillance [3]. Optimal LSFF reach and coverage requires consumption of recommended quantities of fortified foods at a population level. Evidence reveals that consumption of fortified foods substantially improved vitamin A and iodine intakes in Tanzania, Uganda, South Africa and Nigeria [10].

Across sub-Saharan Africa, fortification has centered on widely consumed staple foods such as wheat flour, maize flour, edible oils, and condiments such as sugar, salt and bouillon cubes (i.e., defined as dehydrated blocks of seasoned meat, vegetable, or chicken stock used to add flavor to soups, stews, and other dishes – West Africa only) [3,11,12]. Yet, to date, the availability of iodized salt in East Africa varies widely (i.e.,42–95%) – demonstrating large coverage gaps, despite that by 1996, it was estimated that over 50% of salt consumed in sub-Saharan Africa was iodized [12–15]. Subsequently, fortification of sugar with vitamin A was first introduced in sub-Saharan Africa in 1998 in Zambia, followed by Nigeria and Malawi in 2002 and 2012, respectively. Oil fortified with vitamin A commenced in Malawi in 2000, and subsequently in Uganda in 2004 [16–19]. Advancements in development of standards for large scale wheat and maize flour fortification were notable in three countries – South Africa (2003), Tanzania (2010−11), and Uganda (2003), based on the establishment of mandatory legislation for edible oil, maize flour and wheat flour [20].

### History of LSFF as part of global public health programs

From 1993 to 2011, LSFF efforts in Eastern and Southern Africa Region (ESAR) were spearheaded by three global micronutrient projects funded by the former United Stated Agency for International Development (USAID), in partnership with non-profit organizations and United Nations (UN) bodies, to support the coordination, resource mobilization, establishment of technical working groups, as well as the development of standards, enforcement, and food laboratory capabilities [17,21]. In 2011, for ECSA-HC, development partners’ financial and technical support and country stakeholders’ strong engagement led to a regional food fortification initiative, which conducted feasibility assessments, identified potential food vehicles (i.e., staple foods such as flour, rice, salt, sugar, and oil), as well as enacted and approved regional food fortification standards for salt, vegetable oil, sugar, wheat flour, and maize flour.

On the other hand, Southern Africa Development Community (SADC) countries experienced considerable heterogeneity in terms of when LSFF programming was initiated. While Zambia mandated sugar fortification with vitamin A in 1998, 11 of 16 member states did not have mandatory LSFF legislation until 2018 [22,23]. Since that time period, SADC spearheaded the Regional Food and Nutrition Security Strategy (2015–2025) which included “*promotion of the development/revision of appropriate evidence-based legislation and enforcement of standards on food fortification and bio-fortification” in* Strategic Objective 3 of Priorities, Interventions and Actions [24,25]. In 2020, SADC progressed to the development of Minimum Standards for Food Fortification for four food vehicles – salt, wheat flour, maize flour and oil – and provided guidance on the minimum fortification levels for identified micronutrients, fortificant compounds (i.e., recommended vitamins and minerals), and the fortification levels (e.g., at production or import and at retail) [26].

In ECSA-HC and SADC countries, momentum for LSFF has been spearheaded by global networks and regional bodies. In 2019, the Iodine Global Network (IGN) and UNICEF created a Regional Coordination Mechanism (RCM) comprised of 25 countries- which provided a forum to exchange on LSFF best practices and cross-country learning among ECSA and SADC member states- calling for a LSFF regional road map.

In 2024, Kavle Consulting, in partnership with the former USAID Advancing Food Fortification Opportunities to Reinforce Diets (AFFORD) project, led a situation analysis to ascertain: 1) the status, challenges, and opportunities for LSFF for East, Central and Southern Africa and 2) to summarize the lessons learned and program considerations to accelerate the development of a strategic regional road map for LSFF.

## Methods

From May-June, 2024, nineteen semi-structured key stakeholder interviews were conducted with global regional and country stakeholders inclusive of key fortification experts from United States, non-government organizations (NGO), and multilateral organizations such as United Nations agencies, as well as countries that are members of SADC and ECSA-HC – specifically Botswana, Eswatini, Ethiopia, Kenya, Lesotho, Madagascar, Malawi, Mozambique, and Tanzania (see Table 1). *Ethics statement* This situation analysis was commissioned and approved by the Regional Coordinating Mechanism (RCM) to inform on SADC and ECSA-HC food fortification country and regional program implementation. Ethics committee approval was not sought for this programmatic analysis given the data was used to directly inform on regional and country road maps and activities for LSFF programming. Stakeholders interviewed were from SADC and ECSA-HC countries with active LSFF programs and/or were members of the RCM. Stakeholders were selected by purposive sampling according to the following criteria: 1) direct experience in or familiarity with the history of food fortification in the region/individual member states and/or 2) knowledge of current implementation of LSFF policies and programs in the region and individual member states (see S1 File). A semi-structured interview guide was used to obtain perspectives on assessment of progress, according to the following themes: enabling environment; legislation, policies and standards; regulation and compliance; markets and industries; challenges in the implementation of LSFF programs, inclusive of sub themes on data gaps, monitoring and evaluation, regulatory compliance and enforcement and opportunities for LSFF implementation, specifically on sub themes of social behavior change communication (SBC), private sector, small-scale millers, leadership and governance; food quality and regulation control and sustainability of LSFF programs. In-depth interviews were conducted remotely using Microsoft Teams in English, Portuguese and French. All interviews were audio-recorded and transcribed verbatim prior to analysis. Themes and sub themes were analyzed using Dedoose qualitative software and presented in our findings.

**Table 1:** The SADC and ECSA-HC food fortification alliance*.

SADC	ECSA-HC
1. Angola, 2. Botswana 3. Comoros 4. Democratic Republic of Congo 5. Eswatini 6. Lesotho 7. Madagascar 8. Malawi 9. Mauritius 10. Mozambique 11. Namibia 12. Seychelles 13. South Africa 14. Tanzania 15. Zambia 16. Zimbabwe	17. **Eswatini** 18. Kenya 19. **Lesotho** 20. **Malawi** 21. **Mauritius** 22. **Tanzania** 23. Uganda 24. **Zambia** 25. **Zimbabwe**

* Member states in both regional organizations are **bolded.**

Data extraction using the visualization tools from the Global Fortification Data Exchange (GFDx), was used to compile country-level information on food fortification legislation, standards, and program implementation status [27].

### Conceptual framework

**Fig 1:**
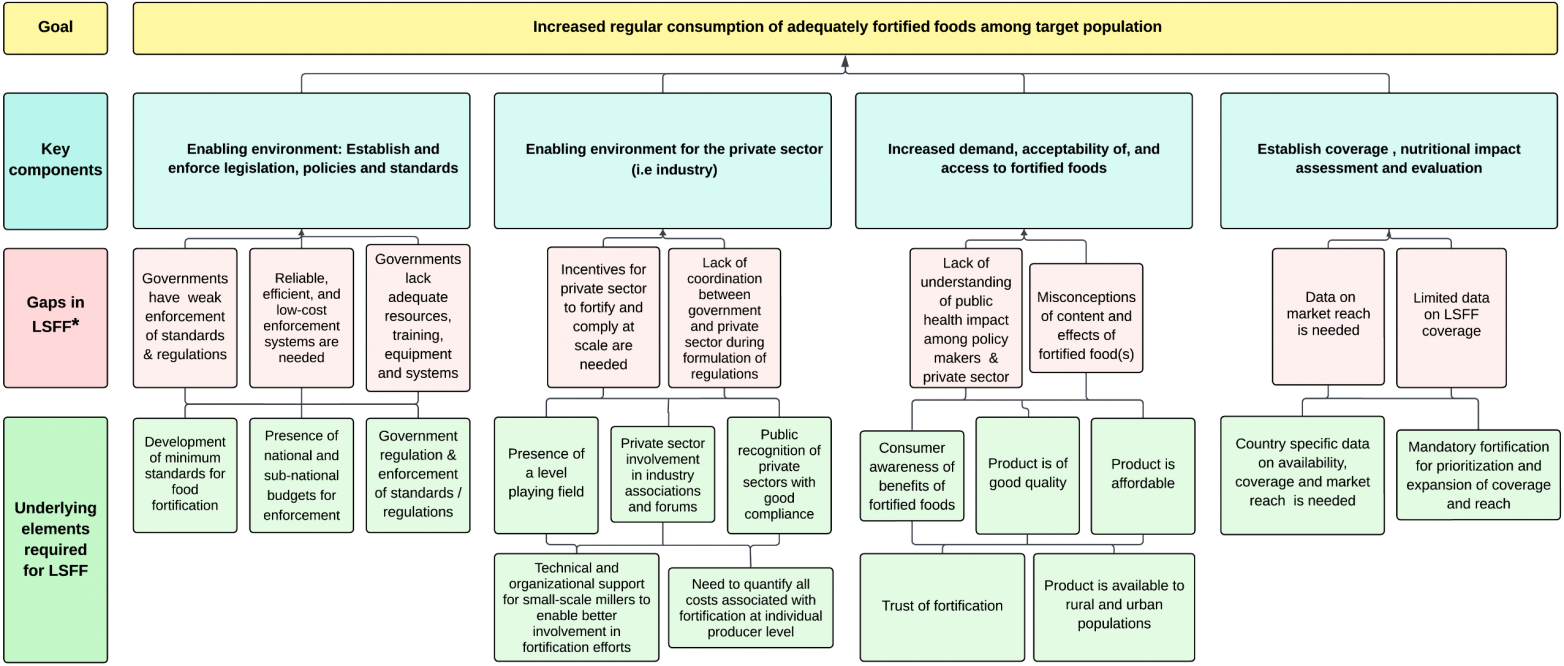
Conceptual framework for food fortification.

The Fortification Blueprint (Darwar et.al., 2023) and Theory of Change Model for Food Fortification (Techout et.al., 2021) were adapted to formulate a conceptual framework (Fig 1) to guide our exploration of the key components, gaps and underlying elements required for LSFF program implementation in ECSA and SADC countries [9,28]. The concepts starred represent data gleaned from this situation analysis.

## Results

### Assessment of progress – LSFF programs

LSFF programs have experienced uneven progress across 18 ECSA-HC and SADC member states- according to the extent of adoption of food fortification legislation (i.e., regulations and standards), the proportion of total food vehicles that are fortified, and/or available data on coverage (i.e., consumption) of fortified foods. Moreover, there is little documentation of LSFF impacts on nutrition and health outcomes.

#### Progress in ECSA-HC and SADC.

**Fig 2:**
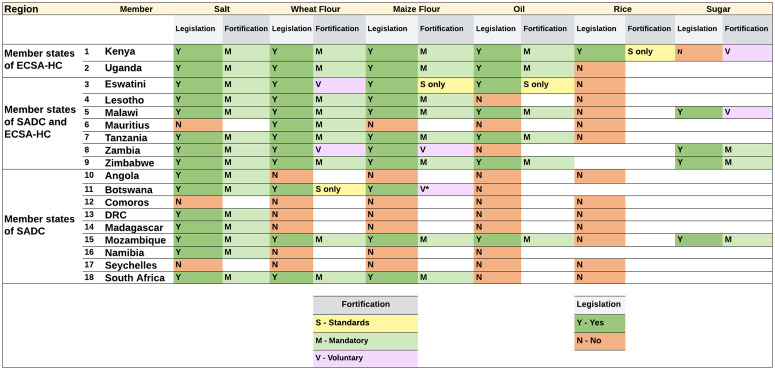
Status of food fortification legislation in member states.

ECSA-HC member states have made greater advancements in LSFF – in comparison to SADC -given most ECSA-HC member states have established legislation on food fortification – (Fig 2) – and collected data on a number of parameters which provide information on the status of fortification of food vehicles (i.e., quantity of total production of the food vehicles, proportion of the food vehicles that are centrally processed – ‘*fortifiable production*’, the percentage of fortifiable foods that are actually fortified, and population coverage of fortified foods) [21].

Notably, Kenya, Uganda, and Tanzania have led the ECSA-HC region in successful LSFF implementation with structured coordination mechanisms, such as annual country-level gatherings and database systems for industry compliance. From 2003–2011, ECSA harmonized fortification standards and monitoring and evaluation at the consumption level with the support of former USAID projects. On the other hand, progress in the SADC countries has been less uniform with variations in legislation, regulations, industry involvement, and laboratory and monitoring capacity to support LSFF. Encouragingly, collaborative efforts with World Food Program (WFP) and United Nations Children’s Fund (UNICEF) aided in developing minimum standards for food fortification within the SADC region. SADC’s annual Food and Nutrition Security Steering Committee has provided a forum to ascertain LSFF progress – with food fortification landscape analyses carried out on wheat and maize flour fortification in Angola, Botswana (+ sorghum), Eswatini (+ edible oil), and Mauritius (wheat flour only) [27].

#### Coverage of fortified foods across ECSA-HC and SADC countries.

**Coverage of iodized salt is high:** Overall, the coverage rate of iodized salt is high among most ECSA-HC and SADC member states, according to data extracted from the GFDx. Most countries have coverage rates of 90% (i.e., South Africa, Eswatini and Zimbabwe, Kenya and Uganda), while few countries showed lower coverage rates, (i.e., 42.5% in Mozambique and 68% in Madagascar). Tanzania and Kenya are the primary manufacturers and exporters of iodized salt to neighboring countries.

**Fig 3:**
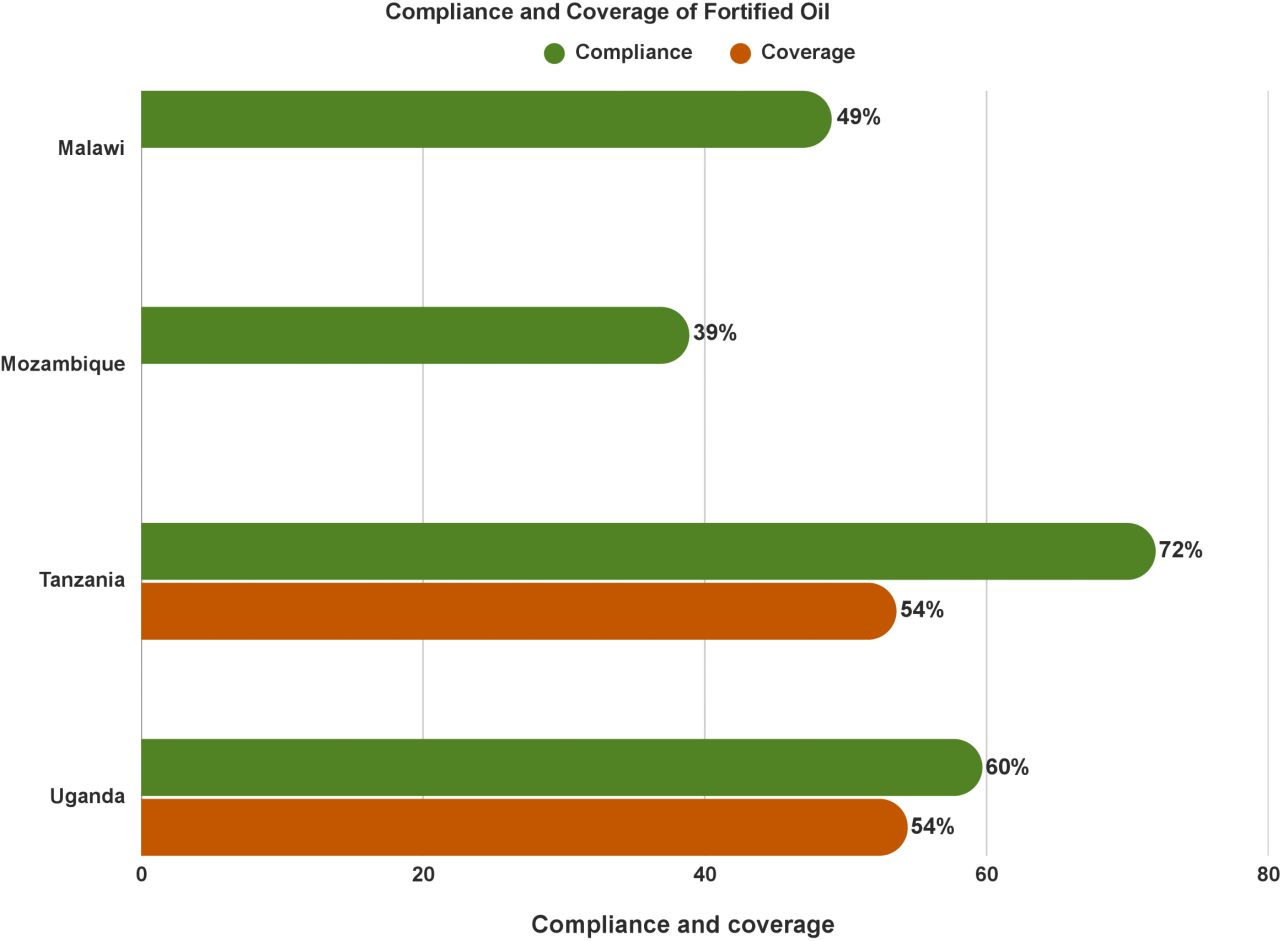
Compliance and coverage of fortified oil, according to GFDx.

**Few data are available on oil fortification:** GFDx has few data on oil fortification SADC and ECSA-HC countries. The compliance to oil fortification standards varied amongst countries- Malawi (49%), Uganda (60%), and Tanzania (72%). Data indicated that coverage of fortified oil reached half of the population in Tanzania (54%) and Uganda (54%) (Fig 3).

**Fig 4:**
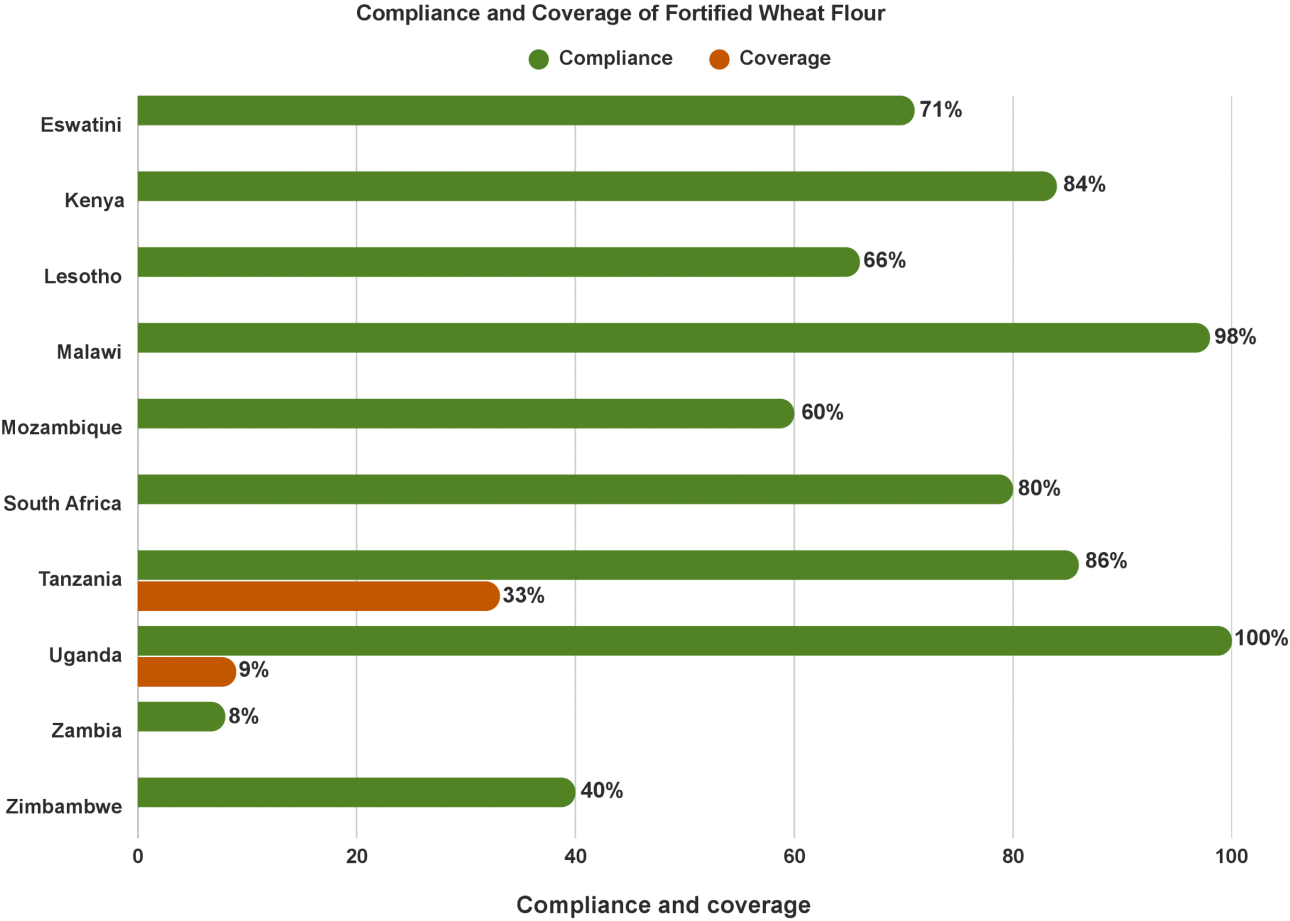
Compliance and coverage of fortified wheat flour, according to GFDx.

**Most wheat flour is centrally produced and fortified, yet consumption is low:** The GFDx dashboard revealed nearly all wheat flour is fortified in Malawi and Uganda, and 60%− 86% of flour is fortified in Mozambique, South Africa, Eswatini, Lesotho, Tanzania, and Kenya. However, since most wheat flour is centrally produced by a few large manufacturers, the percentage of the population who consume fortified wheat flour is low (i.e., urban populations only) and few countries have consumption data, except for – Tanzania (33%) and Uganda (9%). Fig 4 shows the compliance and coverage data among countries with data recorded on GFDx.

**Fig 5:**
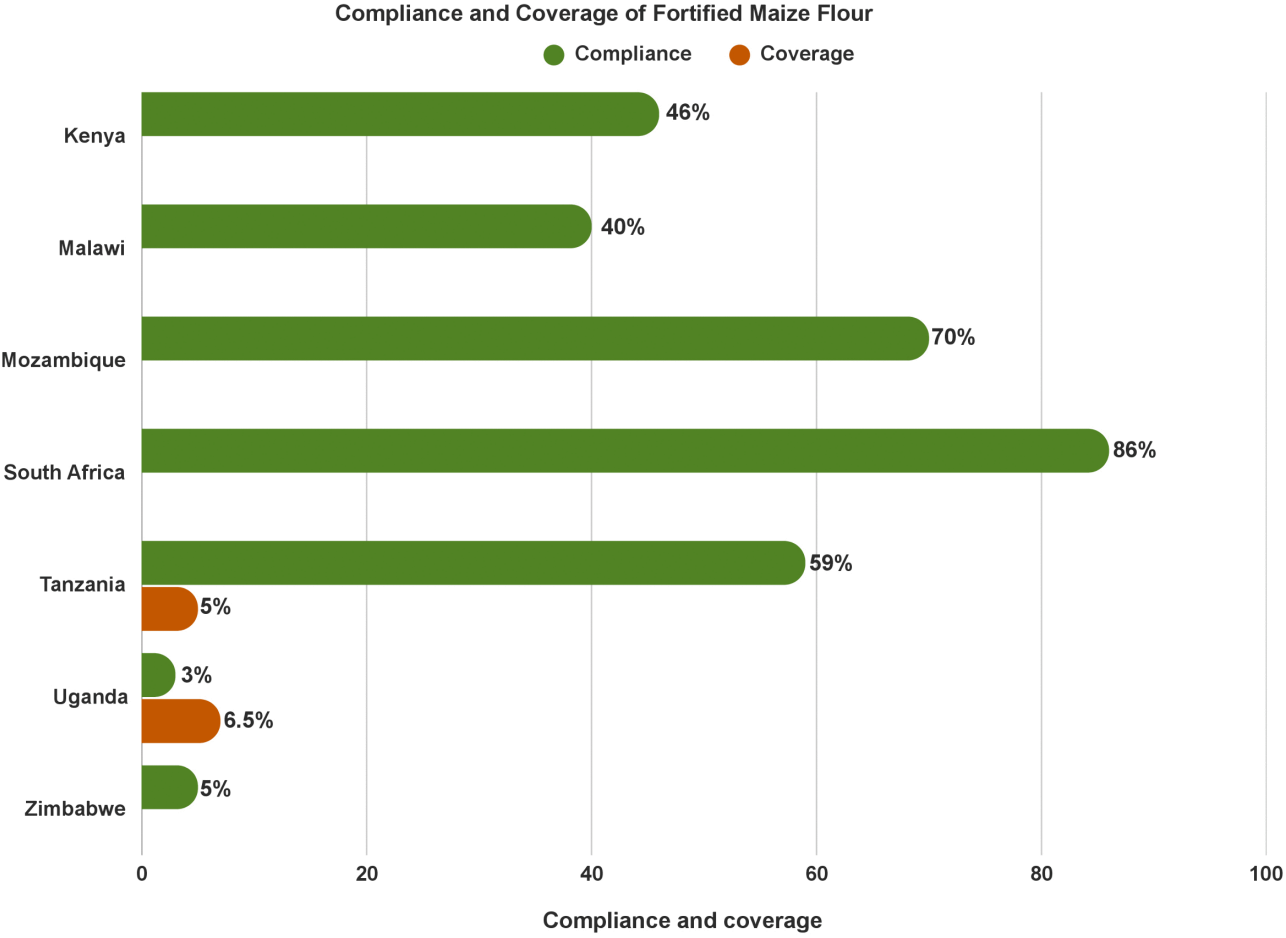
Compliance and coverage of fortified maize flour.

**Compliance of maize flour fortification varies, and coverage is negligible:** The compliance rate of maize flour fortification varied widely from < 5% (i.e.,) Zimbabwe to 70–86% in Mozambique and South Africa. Yet, consumption of fortified maize flour is minimal – e.g., 5% in Tanzania (Fig 5). The majority of stakeholders reported that large scale maize flour fortification is limited in several countries due to the predominance of small-scale milling of maize intended for direct consumption by local consumers.

### Enabling environment for LSFF programs

Elements of a conducive policy environment inclusive of national coordination, prioritization, adoption of standards and policy implementation are discussed below.

#### Legislation, policies and standards.

**Legislation:** Nearly all SADC member states have mandatory legislation on salt fortification as well as legislation on wheat and maize flour fortification (Table 1). Oil and sugar fortification is mandated in Mozambique, Malawi, Tanzania, Zimbabwe, Kenya and only oil in Uganda. In addition, a few countries have standards or implemented voluntary fortification of rice, millet and sorghum fortification.

**Mandatory legislation versus voluntary fortification:** Stakeholders discussed the value add of mandatory fortification versus voluntary fortification as shown in Box 1. Most stakeholders stressed that the regulatory approach to fortification is a critical determinant of LSFF program coverage, industry compliance, and population-level impact.

**Box 1. Mandatory versus voluntary fortification.**

Mandatory fortification occurs when governments legally oblige food producers to fortify foods or categories of foods with specified micronutrients, providing high certainty that these products contain the predetermined quantities of specified micronutrients [29].

When adequately enforced, mandatory fortification can create a business environment where all millers will fortify staple foods, which can push non-fortified products, either domestically produced or imported- which are often sold at lower price points, out of the market. Domestic producers may face difficulties to sell their fortified goods when imported unfortified goods are allowed in the country, as relayed by a global stakeholder below.

*“Mandatory fortification levels the playing field by making everybody fortify. Fortified foods aren’t required to be mandatory in high income countries because people want more micronutrients and consumers can pay a premium for added nutrients. However, in low- and middle-income countries where a penny can make a difference, they must make that difficult decision, if the consumer doesn’t see the difference between an unfortified versus fortified product.”* – Global NGO stakeholder.

Voluntary fortification is defined as addition of nutrients to a food that is not mandated by the government, yet manufacturers choose to fortify their products to increase their brand value [26]. The impetus for voluntary fortification stems from industry’s ability to compete with imported fortified goods on the domestic market and consumers’ preference to purchase fortified products versus the non-fortified products.

**Policy environment supportive of LSFF:** Most regional stakeholders acknowledged that the SADC and ECSA-HC regions have enabling environments that are supportive of LSFF. Yet prioritization, coordination and implementation of policies can hinder LSFF implementation.

Regional and country experiences demonstrated that lack of alignment and coordination between the government and private sector during the formulation of policies, legislation and regulations is a major barrier to policy uptake and industry compliance. As described below:

*“In Mozambique, while fortification is mandatory, there are industries that still don’t understand why and feel forced to comply without being provided with facilitating incentives.”*

*-* Government Stakeholder, Mozambique

On the other hand, since 2005, the Kenya National Fortification Alliance has brought an alliance of health and trade ministries responsible for surveillance and enforcement of the mandatory food fortification standards, cereal millers’ organizations, salt and oil industries, consumer organizations, and development partners – an effective strategy to enhance enforcement and compliance of fortification standards.

**Standards require adoption at country level:** Existing country or regional standards require periodical reviews and updates as some stakeholders discussed that *“[standards] need to be informed by science, local practices and the nutritional status of the population.”* Standards are legally binding only when the producer claims that the product is fortified. On the other hand, if a country does not have their own standards a regional standard may be adopted. For example, other than for salt iodization. Botswana holds no country standard, and plans to adopt the SADC minimum food fortification standards delineated in its first food fortification strategy [30].

From 2003–2011, ECSA-HC worked with partners on a regional effort to coordinate the implementation of food fortification programs- including the harmonization of standards [21]. ECSA-HC drafted the food fortification guidelines and collaborated closely with the Eastern African Community to bring the guidelines into a regional standardization framework and the adoption of the standards through consensus as “*tools of trade*.”

*“Once a common set of standards has been adopted by member states of a regional group, it’s not that complicated to facilitate trade of the fortified food. In the ECSA-HC region, the standards of fortified foods have been harmonized, [which] means all imported and exported goods must meet certain standards.”*

- Regional NGO stakeholder

### Challenges in implementation of LSFF programs

Beyond policies and standards, this review revealed key challenges that affect LSFF program implementation.

#### Lack of accurate and available data to inform LSFF programs.

Data is sorely needed to inform on LSFF programming. The majority of country, regional, and global stakeholders discussed the importance of having up-to-date data on market reach and consumer coverage to ensure that fortified foods benefit the intended population. Most stakeholders relayed concerns around accuracy and/or availability of country-level LSFF data– stating that” i*n terms of key challenges, data on consumption, intake, production or market- is really old.... over 10 years old.”* At regional and global levels, most stakeholders relayed concerns around accuracy and/or availability of recent country data to inform on LSFF programming. Stakeholders expressed frustration that governments and producers don’t “*prioritize it [data] because they are not feeling pressure and nobody’s keeping up with it*.”

Data from micronutrient surveys can determine not only the quantity of fortified foods and micronutrients that are consumed, but also the consumption patterns of fortifiable food vehicles in different population strata (i.e., based on gender, age, geographical location, and economic status). In Mozambique, stakeholders noted that the absence of recent data persists despite earlier plans to strengthen surveillance systems through surveys *“to improve and monitor trends in the national fortification program’s impact.”*

Further, there is a disconnect between methodologies used by industry (i.e., minimum threshold- defined as a set minimum amount of added micronutrients) versus country governments (i.e., use of ranges – defined as minimum to maximum allowable levels of vitamins and minerals added to food products) in sampling the micronutrient content of food – affecting the quality and usefulness of LSFF coverage data.

On the other hand, tools for monitoring fortification production, consumption, and coverage exist, yet require systems in place [31]. Country stakeholders from Mozambique, Ethiopia and Tanzania highlighted the need for monitoring systems to ascertain the level of consumption of fortified foods.

#### Capacity strengthening is required for effective LSFF monitoring and evaluation.

Nearly all stakeholders stressed the need for capacity strengthening for governments, private sector, and regulatory bodies as key to ensuring successful regulatory controls. A global stakeholder emphasized the need for training industries that have not complied with food fortification standards, with a focus on quality control and assurance. Monitoring and evaluation of LSFF programs at country level was perceived to be effective when a collaborative effort between various government ministries and institutions was brokered. For example, Tanzania, has made progress in establishing multiple partnerships to ensure adequate monitoring and availability of household coverage data for LSFF, as described below,

“*The country has established a* *signed contract with regional commissioners for reporting process monitoring indicators biannually to the President’s Office – to ensure high coverage of nutrition interventions at the community level. Through the nutrition information system both process and impact data can be made available and reached by different stakeholders in the country.*

*-*Government stakeholder, Tanzania

#### Regulatory compliance and enforcement.

Despite mandatory fortification in most countries, incentivization of the food industry to fortify and comply at scale remains challenging.

**Compliance by Industry:** In countries with mandatory food fortification, some stakeholders expressed concerns that imported fortified foods are less expensive in comparison to the cost of producing local fortified foods that are compliant at scale. Industry compliance is further hindered by insufficient quality checks at country border points due to limited capacity for inspection and laboratory support for local regulators to ascertain whether imported products meet national fortification standards.

**Enforcement by Governments:** Even for countries with mandatory legislation for food fortification, some stakeholders reported weak enforcement of standards and regulations due to lack of resources, training, and equipment.

*“Zimbabwe has a policy and strategy for fortification with several vehicles [yet] has challenges with enforcement. They have officers who engage with industry to get samples and test. But most of them are not trained, and don’t have the equipment needed for testing. So, you find that the policies are there, the legislation is there and even the strategy, but the things needed for them to work are missing.”*

– Regional stakeholder, SADC

In Eswatini and Lesotho, country stakeholders expressed delays and reduced efforts by government institutions to enforce the relevant legislative actions due to resource constraints. In some member states, e.g., Mozambique, stakeholders relayed that enforcement of monitoring and compliance is donor funded, time-bound and unsustainable in the long-term. Nonetheless, Malawi provides an example of strong enforcement. While about 95% of salt is imported from SADC members states, the Malawi government has adequate regulatory control systems at the border points, which ensures that the unfortified salt is not allowed in the country.

Continuous compliance checks, and enforcement of fortification standards by government authorities is key for long-term success of LSFF programs. Reliable, efficient, and low-cost enforcement systems are needed to avoid an unfair competition of some industries [32]. Government capacities should be improved to not just create regulations, but also to enforce and monitor these regulations. Given there is a dearth of compliance data in the GFDx, countries need to budget for monitoring fortification levels in food samples against the standards.

### Opportunities to improve LSFF program implementation

#### Generation of awareness targeting governments, industry, retailers, and consumers is needed.

Challenges with consumer awareness and trust are thought to negatively impact consumption of fortified foods, according to stakeholder interview data.

Most country stakeholders noted long-term advocacy and integration of iodine awareness in the education system increased the acceptance of iodized salt among communities, especially in ECSA-HC. However, aside from universal salt iodization (USI), the *“knowledge of large-scale food fortification among the general public in the region is too low”* and higher price differential of some fortified food products may pose an affordability challenge for consumers. Both regional and country stakeholders stressed that most consumers are not aware of “*what fortification is*” despite the presence of fortification logo on the product.

*“I think for consumers there is a general lack of awareness of what this is [fortification]... So, you find that the majority of the population know about iodine in salt. I think the education that was done for iodine really worked because it was cascaded through the education system.... I think most young people are aware [of iodized salt], but when you look at the other new [food vehicles] we still have a long way to go...to educate the community in general. Knowledge of large-scale food fortification among the general public in the region is too low and more could be done.”*

-Government stakeholder, Lesotho

Despite fortified foods being indistinguishable from their unfortified counterparts in terms of taste and texture, some stakeholders emphasized the importance of continuous SBC to ensure consumers understand the health benefits of fortified foods versus unfortified foods. Several regional and country stakeholders relayed that some consumers had suspicions of what was added to fortified foods, and “*had to put a lot of public awareness for them to understand”* noting the need for continuous public education as relayed below.

“*I see it as the role of the government to ensure that its citizens get educated through continually engaging with the population to educate them on the benefits of fortified foods.”*

- Regional stakeholder, ECSA

Country stakeholders from Botswana, Tanzania, Malawi, Eswatini and Ethiopia described the need to specifically raise community awareness around LSFF, to strengthen capacity of governments and industry and “*to be deliberate in strengthening the relationship between industry and governments.*” From country stakeholders’ perspectives, implementing changes around awareness creation and increasing advocacy for fortification “*can be used to improve the [knowledge of] nutritional content [of the diet], and people will see the needs [and value of fortified foods]”* which can help create appreciation for fortified foods at both country and regional levels.

In addition, targeted SBC is needed to broaden industry’s understanding of the broader public health population-wide impacts of LSFF. According to regional stakeholders, some large-scale food producers advertise the health benefits of their fortified products, indicating a readiness to align -profit making with the broader public health advantages of LSFF. To enhance engagement with the private sector, some stakeholders suggested increasing awareness among private sector actors on the health benefits of LSFF – demonstrating how even small additions of nutrients can significantly contribute to improved health outcomes.

#### Importance of private sector for LSFF.

All stakeholders underscored the importance of active engagement with the private sector (i.e., millers and food manufacturing industries) across the entire LSFF value chain. While the private sector is primarily motivated by profit, a return on investment for their involvement can be an incentive for covering the cost of fortification. Since the private sector’s motivation often stems from market advantages and brand competitiveness, a multi-sectoral approach incorporating both the private and public sectors is necessary to ensure that LSFF programs are sustainable and effective. Most stakeholders expressed that the private sector often do not allocate time and resources to participate in collaborative LSFF initiatives unless their participation is sponsored. High costs associated with compliance fees and necessary infrastructure investments bore by the industry is a barrier to participation.

Most global, regional and country stakeholders highlighted the need for incentives, such as transferring fortification costs into the product price, and public recognition programs to reward companies for their contributions to public health. Some global stakeholders recommended implementing annual recognition of compliant brands to encourage consumer trust and industry compliance. As expressed by a global stakeholder the mutual benefits of public-private partnerships are “*where the private sector can benefit from increased competitiveness and market share, while [simultaneously meeting] public health goals through improving the nutritional quality of widely consumed foods.”* Some country stakeholders highlighted a critical need for governments’ intervention to create conducive policies and incentives for the private sector. Government mandates on fortification should be supported by policies that facilitate implementation. Without such measures, industries may question the feasibility and necessity of compliance.

#### Engagement of small-scale millers.

Small and medium producers often view fortification as an added financial burden, especially in country contexts with limited government support. Most stakeholders described the operational and regulatory challenges of integration of small-scale millers into LSFF programs. Limited technical capacity, and high compliance burden necessitate tailored approaches from those applied to large-scale millers. Stakeholders emphasized that targeted capacity strengthening, incentives and revision of regulatory frameworks are essential tools for engagement of small-scale millers. Moreover, the additional efforts needed to ensure products by small-scale millers meet fortification standards have high-cost implications to both the government and industry, often leading to sub-optimal implementation of LSFF programs (see Box 2).

**Box 2. Engaging Small- Scale Millers for LSFF: Is There a Value Added?**

LSFF requires equipment, fortificants, and operations that large manufacturers have better resources and abilities to acquire in comparison to micro, small, and medium enterprises (MSMEs). Large companies also have stronger capacities and more experiences to interact with governmental food control authorities in the occasions of inspection and regulatory control [33].

On the other hand, some staple foods, such as maize flour, which are widely consumed in East, Central and Southern African countries, are processed primarily by small millers that may not have the tools or resources to carry out LSFF. In countries where fortification is mandated, small producers risk punitive actions for non-compliance with government standards when their products are not fortified. Small scale millers need support (i.e., technical, financial and organizational) to contribute to country and regional fortification goals.

*“We can use the economies of scale when small producers are together, they can bargain premixes at reasonable prices [and] cost share a food technologist, a food scientist, or an agronomist. They also need the support for quality control and on issues related to packaging, because micronutrients are getting lost in the process from the processing to storage and transportation”*

- Global stakeholder, multilateral organization

Countries such as Tanzania and Mozambique have carried out local efforts to integrate small-scale millers into fortification activities. This has involved mapping and profiling millers and providing necessary machinery and premixes to enable compliance with national and regional nutrition standards. The recognition of the significant market presence of small-scale millers has prompted governments to revisit regulations initially designed for large-scale operations. This shift aims to make fortification practices more applicable across different production scales. While support for small-scale millers may not produce high economic returns, some countries have a strong interest in the promotion and support of small businesses – which often serve as the financial backbone for communities. Additionally, another potential approach to facilitate participation in LSFF is centralized fortification, where small producers’ aggregate food products for batch fortification, to address technical and cost constraints faced by small-scale millers.

#### LSFF as a sustainable, cost-effective intervention.

Countries’ advocacy for inclusion of fortificants in the list of essential medicines allow for tax waivers or reductions. According to several country stakeholders from Botswana and Kenya, LSFF is “*a cost-effective intervention with minimal additional costs*.” Yet, on the other hand, some country budgets do not allocate funding for the implementation of fortification strategies, which affects sustainability. Some country stakeholders proposed that free trade within the region may be a key opportunity to improve food fortification implementation across the region.

*“LSFF is a cost-effective way of supplying the population with recommended levels of micronutrients, but we need to build a case for policymakers to prioritize this intervention among all the health interventions as it is a preventive intervention.”*

-Regional stakeholder, ECSA

## Discussion

### Recap of findings and strengths

To the best of the authors’ knowledge, this situation analyses is the latest progress update on the status, challenges, and opportunities for LSFF programs in the ECSA-HC and SADC regions. LSFF programs have shown uneven progress across the ECSA-HC and SADC based on the extent of adoption of food fortification legislation, the proportion of total food vehicles that are fortified, and/or available data on coverage (i.e., consumption) of fortified foods. ECSA-HC member states have made greater advancements in LSFF – in comparison to SADC – given that most ECSA-HC member states have established legislation on food fortification and collected data on fortification status of food vehicles [8].

Our findings identified key systemic challenges that impede high LSFF coverage of fortified foods and are consistent with published literature, inclusive of lack of LSFF coverage and market data, operational and regulatory issues, weak capacity for regulatory monitoring and enforcement, poor compliance, supply chain and cost constraints for premix, and the need to generate consumer, government and private sector awareness of the health benefits of LSFF [34,35].

The importance of the private sector was underscored in this situation analyses- specifically on the need for incentives, such as transferring fortification costs into the product price, and public recognition programs to reward companies.

### Paucity of LSFF data

Limited coverage data emerged as a key barrier hindering countries’ ability to monitor and evaluate the geographical reach and availability of fortified foods. Most LSFF programs do not report on compliance, coverage, or impact, resulting in gaps in monitoring program effectiveness [36]. Moreover, countries lack data on the market share that large scale producers reach with their fortified products, especially at sub-national levels, which can be used as a measure of progress in fortification. Country-specific data on availability, market reach, and coverage of fortified foods are essential to ascertain whether fortified foods improved the micronutrient nutrition among the intended populations. Mandatory fortification is of utmost importance for countries to prioritize and scale coverage and reach of fortified foods [37].

### Social behavior change: Awareness of the public health benefits of LSFF

This situation analysis found a lack of public awareness around the importance and health benefits of fortified foods despite the presence of a fortification logo on the product– which was of concern, as voiced by country stakeholders. This challenge has been compounded by a preference by large segments of the populations across the African continent, for unfortified maize meal and/or flour, locally produced by small scale millers [8,38]. Behavior change has not been traditionally a part of past LSFF efforts, given the assumption that mandatory fortified foods are widely consumed at the population level. The convenient access and lower prices of unfortified foods from small scale millers vs. higher priced, large scale produced fortified foods, as well as the misperceptions of the ingredients used and lack of perceived benefits in fortified foods warrant explicit consumer LSFF SBC campaigns. Our findings revealed that such campaigns, often implemented jointly by Ministries of Health and industry alliances, have been used in sub-Saharan Africa to inform the public about fortified staple foods and condiments (e.g., iodized salt, vitamin A–fortified oil, or iron-fortified flour), which helped populations identify these products by fortification logos or labels at the point of purchase [39].

Complementing these efforts, clear front-of-pack (FOP) labelling (i.e., that clearly indicate when foods are high in salt, sugar, or fat) will aid consumers’ understanding of the health benefits of fortified food products, while simultaneously prohibiting overconsumption of certain food vehicles that are associated with concerns surrounding increased risk of non-communicable diseases (NCDs) (e.g., sodium in fortified bouillon cubes, saturated fat in fortified edible oils, and free sugars in fortified sugar) [29,40].

### Role and importance of private sector engagement for LSFF

LSFF requires the engagement of the private sector, inclusive of producers, millers, processors, and distributors. Further, LSFF has been increasingly viewed as part of the industrial food value chain which involves supplies, logistics, regulatory systems, marketing, and consumer demand. The Arusha Statement on Food Fortification summarized commitments to address challenges around monitoring, and compliance of LSFF – which pinpointed leveraging co-investment by the private sector alongside governments [41].

A fundamental challenge that affects private sector engagement in LSFF is the various costs associated with fortification – as food industries operate on small profit margins [14]. Additional issues affecting quality of fortified foods in ECSA- HC countries are low compliance to government regulations, weak enforcement, active informal market engagement with SMEs, limited premix supply chains, as well as varying food vehicle consumption, and suspicion and resistance from the public. It is vital to incorporate LSFF in companies’ business strategies or value propositions via branding, consumer segmentation, and premium product lines, which are occurring in similar country contexts [42].

The GFDx, the primary global data platform on LSFF, has reported weak compliance by the private sector in some countries [8]. However, to date, few efforts have been made to quantify the program costs and returns (e.g., financial and reputational risks and benefits) related to fortification – which can identify concrete policy and fiscal incentives to support private sector participation [43]. Moreover, the cost of rolling out LSFF programs – and solutions to absorb all costs related to food fortification – need to explicitly planned and budgeted for among government and private sector stakeholders.

### The role and importance of small millers

While LSFF targets centralized, industrial-level processing, inclusion of small scale millers in national fortification efforts can maximize population coverage and prevent market distortion, especially in countries, where a large proportion of the population consumes an unfortified staple food (i.e., unfortified maize or wheat flour) [44]. Fortifying only products from large mills can lead to unfortified and often cheaper products widely available on the market. Consumers may opt for unfortified and less expensive alternatives – which undermines the effectiveness of LSFF efforts and exacerbates the nutritional disparities between urban (e.g., where industrially milled products are more common) and rural populations who rely on small and medium scale mills as the main source of their staple food supply. To effectively integrate small scale producers, LSFF programs must adopt a blended approach to overcome challenges such as: inadequate access to fortification machinery; lack of skills in fortification techniques, quality control, or business management; lack of understanding of monitoring guidelines; high fines for non-compliance; limited transport, market linkages, and consumer awareness and demand [45,46]. While enforcing mandatory fortification and strict quality control for large industrial mills, countries should develop context-specific, affordable, and practical strategies for small scale and medium mills, such as subsidizing premix, providing simple and robust dosing equipment, and/or organizing pooled purchasing of micronutrient premix with tax exemption – as Africa has one of the highest tax rates for premixes [45]. Offering streamlined training and quality assurance systems and creating cluster-based models (e.g., Fortification Assessment Coverage Toolkit (FACT) surveys to assess quality, coverage, and impact of fortified foods) can go far in monitoring and technical support.

### Limitations

This situation analysis did not encompass an in-depth review of individual country progress in the SADC and ECSA regions, given the timeframe, scope and donor mandate. One key limitation was the absence of private sector perspectives from industry stakeholders who have been involved in LSFF implementation, however the analyses incorporated perspectives on the role of private sector as relayed by other stakeholders.

### Program considerations and future directions for LSFF

Moving forward countries should consider the following elements (Table 2) for successful LSFF program implementation (see Table 2).

**Table 2:** Program considerations for LSFF implementation.

A. Geographical reach and target populations	• Develop and implement robust data collection and reporting systems to gather and report accurate data on market reach and coverage of fortified foods • Integrate reach and coverage data collection tools into existing national nutrition and health surveys to evaluate the health impact of fortified foods • Harmonize indicators to measure market reach and consumer coverage
B. Policy and legislative Action	**•** Leverage existing regional standards and guidelines to formulate country food fortification policies and strategies **•** Define roles and responsibilities to avoid duplication and ensure accountability
C. Leadership & Governance	**•** Leverage existing government institutions and structures to strengthen coordination between stakeholders to advance LSFF programs
D. Supply	**•** Coordinate all stakeholders including industry networks within countries and large-scale producers at the regional level, providing a platform for learning exchange • Improve monitoring at country borders to ensure that imported products are fortified based on set standards **•** Encourage regional free trade to allow large-scale producers to export more fortified foods and motivate local manufacturers to initiate production **•** Formulate standard premix regulations to streamline procurement and increase their availability in countries
E. Consumer awareness	**•** Implement comprehensive and continuous public education initiatives to address the lack of awareness, misinformation and misperceptions about fortified foods **•** Learn from the success of USI campaigns by leveraging multiple platforms, including schools, community outreach programs, and media campaigns to enhance understanding of the benefits of food fortification **•** Allocate sufficient funding to these efforts to enhance public acceptance of new fortified food vehicles **•** Gain a better understanding of consumer behavior, preferences, and barriers to acceptance of fortified foods. **•** Use these insights to tailor interventions and improve communication efforts
F. Food quality and regulation control	**•** Improve governmental food inspection practices and integrate government laboratories with external audits to enhance credibility and reduce the risk of collusion **•** Conduct capacity development for officials, to identify whether fortified and non-fortified products are eligible to be imported into the country **•** Enhance local monitoring teams’ capabilities through regular training and the provision of necessary tools and resources for simple, low cost, but still accurate data collection and timely feedback **•** Simplify the current concepts and practices of industry compliance and governmental enforcement **•** Encourage social auditing programs to complement government inspections and monitoring
G. Private sector involvement	**•** Develop and implement policies that provide public recognition of producers with good compliance **•** Use case studies to demonstrate effective government-private sector collaboration **•** Examine what is needed for small producers to grow and adopt efficient production practices
H. Data gaps	**•** Assess the potential benefit of the micronutrient program, according to presence and cause of deficiency, potential to respond to what is needed for achieving efficacy and effectiveness, and analyze the feasibility and sustainability of the interventions [47] **•** Prioritize and create national and subnational budgets for LSFF enforcement, monitoring and evaluation **•** Estimate the additional intake of micronutrients by vulnerable populations based on the average content of micronutrients in the fortified foods at the consumer level **•** Monitor data collection needs

Moving forward, the RCM can close gaps in country policies and strategies and advocate for adoption of minimum standards (see Box 3), as well as drive regional actions and in the development of regional roadmaps.

**Box 3. Recommendations for the Regional Coordinating Mechanism (RCM) for regional road maps.**

1. Devise a comprehensive assessment for member states, through a multi-sectoral task force consisting of representatives from government, industry, and research, building on this situation analysis and the Global Fortification Data Exchange, covering information with respect to:
  Enforcement and laboratory capacity (i.e., human resources, skills and equipment).
  Production and distribution in geographical regions (in raw ingredient versus in processed product format) and import/export data of main potential food vehicles (commonly adopted versus those unique to a country contexts).
  Custom regulation, tariff and free trade within the region.
  Harmonization of premix composition and standards, and enforcement.
2. Collaborate with SADC standard committee and the Bureau of Standards in each member state to develop a harmonized fortification standard for adoption by all member states.
3. Establish a communication mechanism with designated regional coordinator and country level focal person that engage representatives from relevant government departments (minimally health, agriculture, trade, and custom), industry, research and consumers that will facilitate communications and discussions of issues, opportunities, and solutions.
4. Compile member states priority needs for support by development partners.
5. Establish and strengthen coordination mechanisms between the private sector and other stakeholders through the formation of regional industry associations and forums to ensure awareness of and need for compliance to standards.
6. Encourage and coordinate governments' alignment to standardize the procurement of fortification premixes. Joint importation strategies can reduce costs for producers- reducing additional costs that might deter consumer acceptance.
7. Work with governments to strengthen cross-border collaboration to harmonize fortification standards and ensure consistent quality of imported fortified foods across the SADC and ECSA-HC regions. Regulatory frameworks should be reviewed and updated to monitor and control the fortification of imported foods- ensuring they meet national nutritional requirements.
8. Mobilize partners in nutrition, governments, and incentivize industries across the region to cover the initial basic costs for establishment of food fortification programs and consider consumer contribution for sustainability once operational.

### The way forward for country action around LSFF

Country LSFF policies should be designed to engage closely with the private sector and create a favorable business environment, to address micronutrient inadequacies. Governments can demonstrate their commitment to LSFF by banning importation of unfortified foods and ensuring compliance and quality of the imported premix. It is critical that industry have an understanding of LSFF’s public health benefits for countries and align their profit-making goals with national fortification objectives.

To improve compliance and enforcement of standards, donors, governments, and industry should procure and update laboratory equipment, and strengthen capacities of regulators and industry technicians through ongoing training to address staff turnover. These efforts will enable the private sector food fortification industry to provide accurate production reports, and regulators to effectively monitor products to ensure compliance with fortification standards.

Allocation of national and subnational budgets for LSFF enforcement, monitoring and evaluation processes; alignment of industry and government micronutrient content sampling methodologies to improve quality of LSFF data; and integration of LSFF program indicators into existing national data surveillance systems, are critical elements in achieving accurate, up-to-date data for LSFF.

Further, it is important that south-to-south learnings between countries are shared on practical strategies for streamlining fortification processes and systems, proper use of available resources, modernization of the machinery for more precise dosing and mixing, and updating of national standards, policies and regulations – which can aid in the scale-up of LSFF. Lessons-learned in strengthening compliance - a common weakness in LSFF programming- can be drawn from other regions of the world (i.e., Latin America), which can provide operational insights into roles and responsibilities of various types of LSFF stakeholders, and approaches to local accountability and sustainability of LSFF via development partners, government, the private sector, research community and civil society [48].

Where conditions are not suitable for large-scale food fortification, complementary interventions should be considered to address micronutrient gaps [32]- inclusive of promoting the consumption of micronutrient-rich foods such as animal source foods and biofortified crops, producing specialized processed fortified foods for targeted programs (i.e., humanitarian/ emergency programs), and the use of micronutrient supplements for specific population groups with confirmed deficiencies.

## Supporting information

S1 File Inclusivity in global research. (PDF)
